# The Long Non-Coding RNA ANRIL in Cancers

**DOI:** 10.3390/cancers15164160

**Published:** 2023-08-17

**Authors:** Aymeric Sanchez, Julien Lhuillier, Guillaume Grosjean, Lilia Ayadi, Sylvain Maenner

**Affiliations:** 1 CNRS, Université de Lorraine, IMoPA, F-54000 Nancy, France; 2 SATT Sayens, F-54000 Nancy, France

**Keywords:** LncRNA, ANRIL, *9p21* locus, cancer, gene regulation, epigenetics, competitive endogenous RNA

## Abstract

**Simple Summary:**

The human genome produces various types of RNA molecules. A significant portion of these are long non-coding RNAs (lncRNAs) exceeding 200 nts without an obvious open reading frame. There are more than 200,000 lncRNAs, but we have limited understanding of their functions. LncRNAs can influence gene activity by interacting with proteins or nucleic acids. Some lncRNAs regulate genes involved in cancer, either promoting or suppressing tumor growth. One lncRNA of interest is ANRIL (Antisense Noncoding RNA in the INK4 Locus), which can affect gene expression through different ways. However, ANRIL’s exact role in cancer is complex and varies in different situations, making it a challenging area to study. This review strives to offer a thorough comprehension of ANRIL’s role in regulating genes and its impact on the development of cancer.

**Abstract:**

ANRIL (Antisense Noncoding RNA in the INK4 Locus), a long non-coding RNA encoded in the human chromosome *9p21* region, is a critical factor for regulating gene expression by interacting with multiple proteins and miRNAs. It has been found to play important roles in various cellular processes, including cell cycle control and proliferation. Dysregulation of ANRIL has been associated with several diseases like cancers and cardiovascular diseases, for instance. Understanding the oncogenic role of ANRIL and its potential as a diagnostic and prognostic biomarker in cancer is crucial. This review provides insights into the regulatory mechanisms and oncogenic significance of the *9p21* locus and ANRIL in cancer.

## 1. LncRNAs and Cancers

The human genome is extensively transcribed into thousands of RNAs. A significant part of the resulting transcriptome corresponds to the long non-coding RNAs (lncRNAs) defined as RNAs longer than 200 nts and lacking obvious open reading frames. The number of lncRNAs exceeds 200,000 and the field lacks evidence supporting the functionality of most of them [1,2,3,4]. Compared to mRNAs, lncRNAs exhibit stronger tissue-specific expression and often function in a tissue-specific manner [5]. The broad definition of lncRNAs encompasses a large and highly heterogeneous collection of transcripts that differ at least in their subcellular localizations and functions. Cytoplasmic lncRNAs mainly modulate gene expression by affecting the different stages of mRNA life, including stability and translation, while nuclear lncRNAs mainly associate with the genome or with premature RNAs in synthesis to regulate gene expression and alternative splicing at the chromatin level, respectively [1]. For instance, by recruiting epigenetic writers, chromatin remodelers, transcription and splicing factors, several lncRNAs control the expression of genes nearby or/and distant from their hosting locus (*-cis* and *-trans* activity, respectively) [6,7]. The diversity of activities of the lncRNAs places them at a crossroad between the genomic and the epigenetic regulations qualified as supragenomic regulations. This refers to the genomic information beyond the level of individual genes, such as the non-coding regions of the genome, involved in the modulation of the organization and regulation of the genomic elements [8].

Cellular experiments have shown that lncRNAs are involved in a wide spectrum of biological processes ranging from cell proliferation, apoptosis and nutrient sensing to cell differentiation [9,10]. Consequently, the deregulation of their expression can affect cell homeostasis and may favor the occurrence and/or development of pathologies. This is consistent with the fact that lncRNAs have been associated with key aspects of the cancer biology such as uncontrollable proliferation, evasion of cell death, metastasis and drug resistance [11]. According to “the lncRNADisease 2.0 database”, lncRNAs are associated with 529 pathologies divided into several categories, including three major ones corresponding to cancers (44.2%), cardiovascular pathologies (11.6%) and neurodegenerative diseases (7.3%) [4]. For these reasons, several lncRNAs are already used as biomarkers as their expression rate correlates with the diagnostic nature of certain pathologies and more importantly they definitively may prove to be valuable therapeutic cancer targets [12,13].

Cancer is one of the leading causes of death worldwide and refers to a group of diseases characterized by the uncontrolled growth and spread of abnormal cells capable or not of invading nearby tissues and organs. Aberrant expressions and splicing profiles of lncRNAs have been found in various types of cancer in agreement with the fact that some lncRNAs act as oncogenes, promoting cancer development and progression, while others act as tumor suppressors, inhibiting cancer growth and metastasis [14,15]. For example, the lncRNA HOTAIR (HOX Transcript Antisense Intergenic RNA) has been found to be overexpressed in several types of cancer and promotes metastasis by interacting with chromatin-modifying enzymes and promoting epigenetic changes [16]. In contrast, the lncRNA GAS5 (Growth Arrest-Specific 5) has been shown to be downregulated in cancer and acts as a tumor suppressor by inhibiting cell proliferation and promoting apoptosis [17].

In this review, we focused on the implication of the lncRNA ANRIL (Antisense Noncoding RNA in the INK4 Locus, CDKN2B-AS1) in cancers. ANRIL is transcribed from the *9p21* locus in several linear and circular spliced variants mainly made of repetitive elements derived from LINE, SINE and LTR sequences [18,19,20]. The overexpression of some of them is correlated with pathologies such as cardiovascular disease (CVD), type 2 diabetes (T2D) and cancers [21,22].

ANRIL can modulate gene expression at the post-transcriptional level by acting as a competing element RNA (ceRNA) of miRNAs and proteins [23,24]. Additionally, ANRIL affects gene expression at the chromatin level negatively and positively by guiding the recruitment of chromatin modifiers or transcriptional activators at specific loci [25,26]. Recently, converging data showed that ANRIL is also able to affect the patterns of alternative splicing in HEK293 and HUVEC cells [20,27]. These regulatory activities are often prone to enhancing cell proliferation, migration, invasion, and metastasis, and to suppressing apoptosis and senescence mainly attributed to the modulation of the expression of key cancer-related genes involved, for instance, in the p53 axis. To our knowledge, ANRIL is the lncRNA with the highest frequency of alterations in the context of cancer development and progression.

The proposed roles of ANRIL in cancer biology are still unclear. This is mainly due to differences in the types of cancer studied and/or the methods used to evaluate ANRIL expression likely to exclude the functional contribution of the individual ANRIL isoforms for instance. It is also possible that ANRIL has different activities in different stages of cancer progression or in different cell types. This drastically complicates the understanding of the mechanisms through which ANRIL promotes cancer development. In this review, we addressed these points and made efforts to formalize a clearer vision of the intertwined domain related to ANRIL and cancers.

## 2. ANRIL and the *9p21* Locus

### 2.1. Discovery of the 9p21 Locus as a Region of High Interest

The *9p21* locus is located on the short arm (p) of chromosome 9 at region 21 and spans over 170 kilobases (kb). This locus has drawn particular attention due to the frequent observation of homozygous deletions or epigenetic modifications, such as DNA methylation-induced transcriptional silencing resulting in the inactivation of this genomic region in multiple cancer types. Additionally, single-nucleotide polymorphisms (SNPs) within this region are linked to several age-related disorders, CVD and cancers. These SNPs are predominantly found within the genes themselves and the adjacent gene desert region spanning approximately 0.3 megabases [28]. Note that the precise mechanisms through which these SNPs exert their effects remain largely unknown.

The *9p21* locus was initially described as containing the *CDKN2A*/*ARF* and *CDKN2B* genes which encode three proteins involved in cell cycle regulation: the p14/ARF, p16INK4a/CDKN2A and p15INK4b/CDKN2B proteins, respectively [29]. Initially, the *CDKN2A*/*ARF* gene was found to be frequently inactivated or mutated in melanoma and other cancers, at least in part due to its ability to modulate the p53 axis [30]. Further investigations allowed researchers to link the *9p21* locus to various other abnormal situations, including atherosclerosis, T2D, Alzheimer’s disease, lupus erythematosus, epilepsy, glaucoma, obsessive compulsive disorder and sepsis [31,32,33,34,35,36,37,38]. The specific genetic variants and mechanisms underlying these associations are complex and continue to be the subject of ongoing research.

Pasmant and colleagues made a significant finding in 2007 by identifying the long non-coding RNA ANRIL encoded within the *9p21* locus. It was promptly recognized as a critical factor in controlling the expression of this locus [31]. As previously stated, this review is directed towards the association between ANRIL and cancers. For comprehensive insights into ANRIL and its involvement in other pathologies, we suggest referring to the reviews authored by Hannou et al., 2015 and Kong et al., 2018 for instance [22,39].

### 2.2. The lncRNA ANRIL and the Genes of the 9p21 Locus

As mentioned above, the *9p21* locus includes the gene that encodes the long non-coding RNA ANRIL. This gene is orientated in the opposite direction (antisense) to the genes within the locus and extends over a region of 126 kb, covering the *CDKN2B* gene located within its first intron. The intergenic region separating the *ANRIL* gene from the *CDKN2A*/*ARF* gene is a bidirectional promoter long to approximately 300 bp [31,40,41].

**Figure 1:**
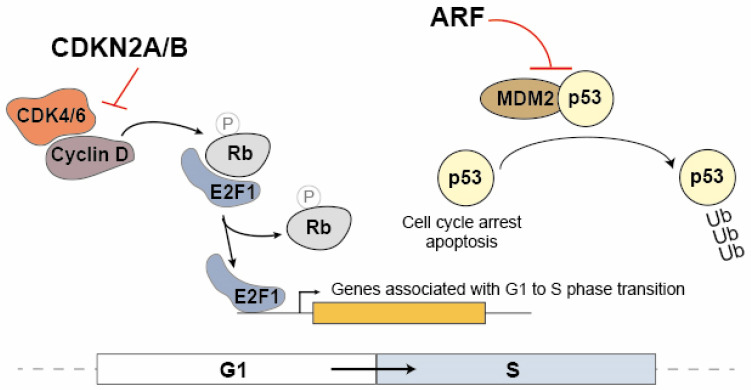
The regulatory mechanisms involving CDKN2A, CDKN2B and ARF on the cell cycle progression. In the G1 phase, the Rb protein (Retinoblastoma Protein) binds to the E2F1 transcription factor, sequestering it. Upon transitioning to the S phase, the CDK4/6 and cyclin D complex phosphorylates the Rb protein, resulting in the release of E2F1. Consequently, E2F1 becomes active and promotes the transcription of genes involved in the transition to the S phase. CDKN2A/2B inhibits the association between CDK4/6 and cyclin D, therefore acting as cyclin inhibitors to control cell cycle progression during the G1/S phase. Additionally, ARF plays a role in cell cycle modulation by promoting the dissociation of the MDM2 ubiquitin ligase from p53, leading to p53 stabilization. This stabilization activates cell cycle arrest at the G1/S barrier.

The CDKN2A and CDKN2B proteins are cyclin inhibitors that regulate cell cycle progression in the G1/S phase by inhibiting the association between CDK4/6 and cyclin D (Figure 1). In the G1 phase, the Rb protein (Retinoblastoma Protein) sequesters the E2F1 transcription factor. During the transition to the S phase, the Rb protein is phosphorylated by the CDK4/6 and cyclin D complex, leading to the dissociation of E2F1. As a result, E2F1 becomes capable of activating the transcription of genes associated with the transition to the S phase [29,42]. ARF also contributes to cell cycle modulation by promoting the dissociation of the MDM2 ubiquitin ligase from p53, leading to its stabilization and consequently to the activation of the cell cycle arrest at the G1/S (Figure 1) [43]. These proteins play a crucial role in regulating the cell cycle, and any dysfunctions in their expression may have significant implications for cancer. Given that ANRIL can modulate the expression of multiple genes, including *CDKN2B*, it is compelling to consider ANRIL a critical contributor to many of the pathological processes dependent on the *9p21* locus.

## 3. ANRIL Expression and Abundance

As the vast majority of the lncRNAs, ANRIL expression is modest (less than 1000 copies per HEK293 cells) and tissue-specific [44,45]. ANRIL is also transcribed by the RNA polymerase II and processed through the canonical splicing, capping and polyadenylation pathways. The transcriptomic analysis of publicly available data by the GTEx consortium revealed that among 53 normal human tissues, ANRIL is more expressed in transverse colon, pituitary glands, small intestine and testis compared to the other analyzed tissues [46]. Beside tissue-specific expression, the ANRIL rate also appears to be dependent on the development stage. For instance, ANRIL is expressed from the toddler stage in testis (2–10 years) [47].

Since ANRIL is crucial for maintaining cellular homeostasis, the regulation of its expression is a very sensitive issue [48,49]. First, it has been shown that the *9p21* locus is contained within a single topologically associated domain (TAD) [50,51]. This specific arrangement depending at least on the CDKN2A promoter and 3 CTCF binding sequences within CDKN2B exon-1, ARF exon-1b, and CDKN2A exon-3 is likely to play a critical role in regulating the expression of the *CDKN2A*/*ARF*, *CDKN2B*, *ANRIL* genes in HUVEC cells and in the colon-cancer-related cell lines GES1, BGC823 and H1299 [50,52,53]. A recent study has demonstrated that the formation of this TAD relies on RNAs and CTCF, with both actors being essential for strengthening TAD insulation, facilitating interactions between enhancers and promoters, and promoting gene expression within the *9p21* locus [54].

ANRIL expression is regulated by multiple factors and stimuli-dependent mechanisms such as genotoxic stress and inflammatory response [26,41,52,55]. For instance, DNA damage activates the E2F1 transcription factor, leading to increased ANRIL expression and subsequently cell proliferation upon DNA repair [41]. HUVEC cells treated with TNF-α or IFNγ (interferon γ) show induction of ANRIL expression by NF-kB and STAT1 activation, respectively, both capable of associating response elements located within its promoter [26,52]. In retinoblastoma, hypoxia induces the direct HIF-1α binding to the ANRIL promoter region to transcriptionally activate its expression [56].

As mentioned before, the bidirectional promoter of the *CDKN2A*/*ARF-ANRIL* genes contains DNase I hypersensitivity regions and CpG islands. Interestingly, the methylation of these elements has been shown to inhibit the association of CTCF, resulting in a decrease in ANRIL expression associated with H3K4me3 reduction [57]. Note that validating a causal link between DNA methylation of the CDKN2A/ARF promoter and ANRIL transcription requires further clarification and could benefit from methods including CpG mutagenesis, reporter assays, and/or epigenetic editing techniques [58].

Conversely, the interaction between ERα (Estrogen Receptor Alpha) and its putative binding site located in this promoter region is facilitated by CpG methylation, resulting in increased ANRIL expression upon β-estradiol treatment of SW872 cells [59]. The methylation status of the ANRIL promoter region also influences the association of the transcription factors SMAD3/4, thereby affecting ANRIL expression levels in SaOS-2 osteosarcoma cells [60]. Additionally, TET2 (Tet Methylcytosine Dioxygenase 2), a tumor suppressor factor responsible for CpG demethylation, decreases ANRIL expression, leading to the inhibition of gastric cancer cell growth [61].

ANRIL expression is further modulated by the oncogenic transcription factors c-MYC, SOX2, and SP1 in lung, pharynx, and liver cancers, respectively [62,63,64]. Moreover, ANRIL expression is likely to be repressed by different factors, including Androgen Receptor (AR) and Phospholipase D (PLD) in prostate and lung cancer, respectively [65,66]. Finally, in the context of colon cancer, when HCT-8 and HCT116 cells were treated with Qingjie Fuzheng Granule (QFG), it was found that ANRIL expression decreased together with TGF-*β*1, phosphorylated (p)-SMAD2/3, SMAD4, and N-cadherin reduction [67].

Additionally, post-transcriptional mechanisms govern ANRIL abundance. In the context of colon cancer, it has been proposed that the association between AUF1 and ANRIL may lead to a detrimental effect on ANRIL stability and that the presence of P14AS lncRNA reduces the interaction between AUF1 and ANRIL. This competitive binding of P14AS lncRNA to AUF1 leads to an increase in ANRIL expression level [68]. In the same line, ANRIL is stabilized by IGF2BP3, favoring proliferation and metastasis in renal cancer tissues and related cell lines [69].

## 4. ANRIL Phylogeny, Evolution and Transposable Elements

LncRNAs exhibit lower levels of conservation compared to protein coding genes, and ANRIL is no exception to this trend. Through a phylogenetic analysis involving 27 species (from zebrafish to human), it was determined that ANRIL originated in placental mammals with a limited number of exons. As a result of specific evolutionary processes, an increased number of exons became evident within the haplorhines cladus, while exons’ erosion was observed during rodent evolution, limiting the use of murine models to study the association between ANRIL and cancers. The primate-specific expansion was attributed to the insertion of transposable elements (TE) within existing exons or introns, resulting in the modification of its functional capabilities [70]. Indeed, the evolution of the lncRNAs is a complex process that is influenced by a variety of factors, including TEs, which are DNA sequences that have the ability to move through a genome [71]. They make up a significant portion of many genomes, including those of humans in the range of more than 60% [72]. Interestingly, the proportion of TEs positively correlates with the number of lncRNA genes, suggesting that the generation and the evolution of the lncRNA genes highly depend on the presence of the TEs [72]. The latter have the ability to insert themselves into the genome, which, via exaptation, may lead to the creation of new transcription units by providing transcription factor binding sites or transcriptional start sites [73]. The resulting “junk RNA” produced via pervasive transcription may serve as a source for the evolution of lncRNA through non-adaptive or neutral processes. Over time, the lncRNA gene repertoire may undergo genome modifications that promote the acquisition of protein/DNA/RNA binding sites, thus conferring relevant functional capabilities to the resulting lncRNAs. These newly evolved lncRNAs can be recruited as new components of existing biological systems or may gain genetic elements controlling its expression in a tissue-specific manner [74]. This is consistent with the Repeat Insertion Domains of LncRNAs (RIDLs) hypothesis, which suggests that these repeat insertion domains act as functional RNA domains through two distinct mechanisms: a specific secondary structure that mediates (1) interaction with proteins, and (2) hybridization to nucleic acids [75]. Interestingly, TEs cover 35% of the ANRIL sequence, and the Exon8, which is 70% covered by the subcategory of LTR named ERVL-MaLR, has been described to be involved in ANRIL genomic occupancy [19,20]. Consequently, it appears that ANRIL adheres to the RIDL hypothesis.

## 5. ANRIL Exons and Isoforms

**Figure 2:**
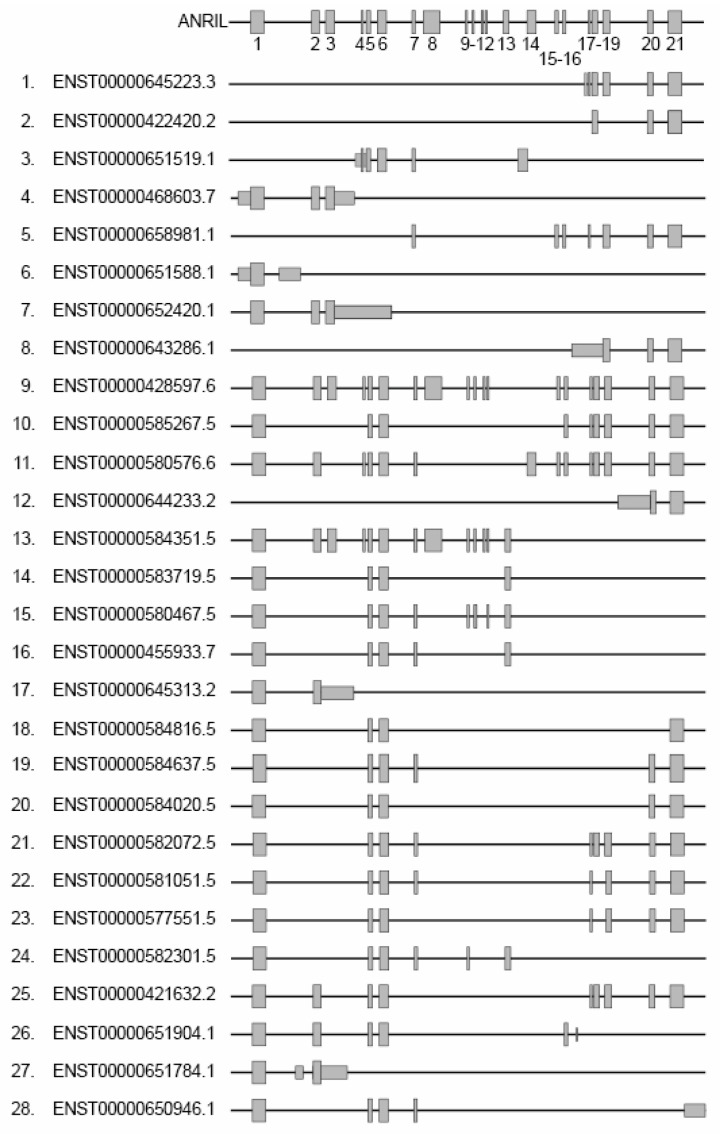
Representation of the different linear isoforms of ANRIL. Grey rectangles represent exons, numbered from 1 to 21. The left side displays isoform accession numbers from the Ensembl database along with alternative nomenclature ranging from 1 to 28.

The *ANRIL* gene consists of 21 exons, with lengths ranging from 74 to 696 nucleotides (mean length of 202 nucleotides) (Figure 2). These exons undergo alternative splicing (AS), resulting in the generation of at least 28 different linear isoforms with varying lengths [76] (from 602 to 7713 nts). To date, the specific mechanism that regulates the alternative splicing of ANRIL is largely unknown. Only one recently published study proposes that m6A post-transcriptional modifications, which involve the addition of a methyl group to RNA molecules, may play a role in the regulation of ANRIL AS. These modifications could facilitate the recruitment of splicing regulators such as SRSF3, which contribute to the splicing process in pancreatic cancer [77].

Figure 2 shows each linear isoform that we numbered from 1 to 28 in addition to the Ensembl accession numbers. Out of the 21 exons, exons 1, 5, and 6 are present in the majority of linear isoforms. These isoforms can be categorized into two groups based on their 3′ extremities and exon composition: the isoforms that include both exons 1 and 21 (isoforms 9–11, 18–23 and 25) or exons 1 and 13 (isoforms 13–16 and 24). When studying a melanoma cell line, it was found that the inclusion of exons in the isoforms varied, indicating a heterogeneous expression pattern. This suggests that multiple isoforms, possibly specific to different tissues, coexist within the cell with potential variable activities [44,45,77,78]. This is consistent with the fact that lncRNAs often consist of multiple exons arranged for creating distinct modules. As an example, the well-known lncRNA HOTAIR implicated in gene regulation and chromatin remodeling acts as a scaffold to recruit two distinct chromatin-modifying complexes and modulate proliferation in cell glioblastoma [79]. In this line, the ANRIL isoforms 9 (ENTS0000428597.6 or NR_003529) and 13 (ENTS00000584351.5 or DQ485454) have been recognized as regulators of gene expression with antagonistic roles. This duality in their functions has been observed in human endothelial cell lines in the context of CVD. When the isoform 9 is overexpressed, it leads to the down-regulation of the *EZR* and *CXCL11* genes, as well as the up-regulation of *TMEM106B*. Conversely, overexpression of the isoform 13 has the opposite effect on these genes. These antagonistic activities are also evident at the cellular level of physiological processes. The expression of the isoform 9 appears to promote transmigration and cellular adhesion, while the isoform 13 elicits contrasting effects [80].

In addition, at least 30 circular isoforms have been identified, which include exons 4 to 16. Circular RNAs (circRNAs) are a type of lncRNA that are abundant, evolutionarily conserved, and often exhibit tissue-specific expression patterns. They are generated through a process called back splicing, and possess unique properties such as resistance to exonuclease degradation due to their lack of 5′ and 3′ ends [81]. CircRNAs can act as competitive endogenous RNA (ceRNA) by sequestering one or multiple RNA-binding proteins (RBP) or miRNAs [82]. Analysis of the ratio between circular and linear ANRIL isoforms within a cohort of patients with CVD and melanoma revealed a significant difference compared to the control groups [78,83]. In addition, Holdt and colleagues discovered a circANRIL, which exhibits anti-atherosclerotic properties by sequestrating PES1, a factor involved in the assembly of the 60S ribosomal subunit. The retention of PES1 promotes apoptosis and halts cell proliferation, both of which are functions associated with the development of atherosclerosis [24]. These findings suggest that the linear/circular ratio is inversely correlated with the progression of the disease, indicating an antagonistic relationship between these two isoform categories. Hence, maintaining an accurate equilibrium in the expression of ANRIL isoforms is believed to be deemed vital for appropriately modulating gene regulation, whereas an imbalance in their expression may profoundly impact cell physiology. Future investigations focusing on the comprehensive characterization of ANRIL isoforms in different cancer subtypes hold the promise of providing crucial insights into the contribution of ANRIL in cancer development.

## 6. ANRIL and Cancer

### 6.1. ANRIL Expression in Cancer

ANRIL has undergone extensive analysis, with researchers employing various methodologies such as RTqPCR, RNA sequencing, or microarrays across numerous cancer cell lines and tissues. In an effort to gain clarity, a review of the literature has been performed and 76 articles have been sorted based on several criteria, such as the accessibility of primer sequences employed in RTqPCR, to compile a comprehensive inventory of the ANRIL isoforms identified in each investigation. The results are indicated in Table 1 and demonstrate that the vast majority of the linear ANRIL isoforms (1, 2, 4, 6, 7, 9–11, 13–28) are upregulated in a wide range of cancer types, including lung (LC), gastric (GC), breast (BC), ovarian (OC), cervical (CC), colorectal (CRC), bladder (BladC), thyroid (TC), brain (BrC), osteosarcoma (OS), myeloma (MM), prostate (PC), leukemia (ATL/AML), melanoma, endometrial (EC), renal (RC), retinoblastoma (RB), head/neck (HNSCC/LSCC), intrahepatic cholangiocarcinoma (iCCA) and hepatocellular (HCC) cancers. The overexpression of ANRIL is associated with poor prognosis and a lower overall survival in GC, LC, HCC, HNSCC/LSCC, RC, iCCA, EC, AML, OS, CC and OC (Table 1). One meta-analysis published in 2022, based on a sample of 1708 cancer patients extracted from 23 studies across three databases, could also established a clear correlation between high ANRIL expression, adverse overall survival rates, larger tumor size, advanced TNM stage, and lymph node metastasis [84].

As mentioned before, it is possible that ANRIL has different activities in different stages of cancer progression. This is consistent with the fact that ANRIL expression is significantly correlated with a higher TNM stage of cancers, including LC, GC, HCC, LSCC, OS, CRC, CC, OC, HCC, BladC and OS (Table 1). In addition, while five articles demonstrate upregulation of ANRIL in non-small cell lung cancer and adenocarcinoma (NSCLC/LUAD) [48,66,85,86,87], one study reports the opposite effect in idiopathic pulmonary fibrosis (IPF), an independent risk factor of lung cancer with NSCLC being the main pathological type [88,89,90]. These differences may arise from variations in the analyzed tissues (NSCLC/LUAD vs. IPF) but possibly from the detection of distinct subsets of ANRIL isoforms too. Indeed, in NSCLC and LUAD, isoforms 1, 2, 4, 5, 7–13, 17, 19–23, and 25–27 were identified, whereas in IPF, only the detection of isoforms 3, 9, 11, 13, 15, 16, 19, 21, 22, 24, and 28 was performed. In consequence, an abundance of the isoforms 3, 15, 16, 24, and 28 was exclusively evaluated in IPF, suggesting that these isoforms may be downregulated in this condition. This observation is in favor of the existence of separate functional units within ANRIL isoforms, which may contribute to different activities involved in the physiological and pathological outcomes of lung-related conditions.

Altogether, these data strongly suggest that the majority of the linear ANRIL isoforms likely possess tumor-promoting capabilities. Since linear isoforms are mainly nuclear, one reasonably hypothesizes that ANRIL over-expression generates excess ANRIL molecules capable of reinforcing the regulatory activity it exerts on its direct targets or even on additional genomic regions. The molecular aberrations thus generated, including, for instance, inappropriate gene modulation, could be responsible for the appearance and/or reinforcement of pathological processes. In agreement, cell line experiments have shown that a reduction in ANRIL leads to increased apoptosis and senescence while decreasing cell proliferation, invasion, and migration.

To conclude, note that the relative levels of circular isoforms of ANRIL have not been systematically explored in most studies. Only two articles have reported an upregulation of circular ANRIL isoforms in cancer, specifically in CC and melanoma [78,91]. Although their association with cancer remains somewhat poorly assessed, it is conceivable to hypothesize that these circular forms may exert a tumor-promoting influence akin to linear isoforms. This may contrast with findings in CVD and T2D, where linear and circular forms of ANRIL have been described to exhibit opposing activities [24,92].

**Table 1:** Summary of the ANRIL expression associated with cancers. It includes information on cancer types, samples used in the studies, the methodologies used for ANRIL detection, the ANRIL isoforms detected, and the corresponding references.

Cancers	ANRIL Expression	Tissues	Cell Lines	ANRIL Detection RTqPCR (Fw_Rv), RNAseq, Microarrays	Detected Isoforms	References
LC	Up	1/87 NSCLC tissues, 2/TNM I stage LUAD	A549, H460, H1299, H1975, SPC-A1, H1650	Ex17_Ex18, Ex1_Ex2, Ex20_Ex20, Ex12_Ex16	1, 2, 4, 5, 7, 8, 9, 10, 11, 12, 13, 17, 19, 20, 21, 22, 23, 25, 26, 27	[48,66,85,86,87]
	Down	24 IPF		Ex6_Ex7	3, 9, 11, 13, 15, 16, 19, 21, 22, 24, 28	[88]
GC	Up	1/20 paired GC, 2/19,317 GC patients and Lymph nodes, 3/83GC, 4/120GC	AGS, BGC823, MGC80–3, MKN-45, SGC-7901, HGC-27, HSC-39, FU97	Ex1/2_Ex2, Ex1_Ex2, Ex11/12_Ex13, Ex1_Ex1, Ex14_Ex15, RNAseq	4, 6, 7, 9, 10, 11, 13, 14, 15, 16, 17, 18, 19, 20, 21, 22, 23, 24, 25, 26, 27, 28	[93,94,95,96,97,98]
BC	Up	1/787 early BC patients, 2/37 TNBC	MCF10A, MCF7, T47D, MDA-MB-231, BT549, HS578T, SKBR3, BT474, BT20	Ex12-Ex15, Ex12_16, Ex5_Ex6/7, Ex3_Ex4, Ex1_Ex1, Ex17/18_Ex18, Hs01390879_m1 (Ex1_Ex2), RNAseq	1, 3, 4, 6, 7, 9, 10, 11, 13, 14, 15, 16, 17, 18, 19, 20, 21, 22, 23, 24, 25, 26, 27, 28	[31,99,100,101,102,103,104]
OC	Up	1/18 OC, 2/86 OC, 3/102 EOC tissues, including 68 SOC tissues	SKOV3, OVCAR3, HO-8910, SKOV3, A2780, Hey, OVCA429, OVCA433	Ex21_Ex21, Ex1_Ex2, Ex4_Ex5	1, 2, 3, 4, 5, 7, 8, 9, 10, 11, 12, 13, 17, 18, 19, 20, 21, 22, 23, 25, 26, 27	[105,106,107,108]
CC	Up	41 high-grade squamous intraepithelial lesions (HSILs), and 75 cervical cancer tissues	CaSki, SiHa	Divergent Ex2_Ex4	CircRNA	[91]
	Up	53CC	HeLa, CaSki, SiHa, ME-180, H1299	Ex1_Ex2	4, 7, 9, 11, 13, 17, 25, 26, 27	[86,109]
CRC	Up	20 from CRC, 20 from adenomatous polyp patient, 172CC		Ex1_Ex1, Ex3_Ex4	4, 6, 7, 9, 10, 11, 13, 14, 15, 16, 17, 18, 19, 20, 21, 22, 23, 24, 25, 26, 27, 28	[67,110]
	Down	Meta-Analysis from 10 sets of RNAseq, 40 patients with CC (with 10 patients each in stages I, II, III and IV)	Caco2	Ex1_Ex1, RNASeq	4, 6, 7, 9, 10, 11, 13, 14, 15, 16, 17, 18, 19, 20, 21, 22, 23, 24, 25, 26, 27, 28	[111,112]
BladC	Up	1/30 BC, 2/51 BC	EJ	Ex1_Ex1, Ex1_Ex2	4, 6, 7, 9, 10, 11, 13, 14, 15, 16, 17, 18, 19, 20, 21, 22, 23, 24, 25, 26, 27, 28	[113,114]
	No misregulation	85 NMIBC	97-1, 97-7, MGH-U3, MGH-U4, RT112, RT 4, UMU-UC5, UMU-UC7, VM-CUB1, 5637	Ex17/18_Ex18	1, 9, 10, 11, 21, 25	[115]
TC	Up	510TC, 502TC	TPC-1, HTH83, FTC-133	Ex2_Ex3, RNAseq	4, 7, 9, 13 (+RNAseq)	[116,117]
BrC	Up	1/15G, 2/142G, 3/10 all stages each, 4/19G	A172, LN18, T98G, U251, LN229, U87	Ex9_Ex12, Ex1_Ex2, Ex13_Ex13, RNAseq/uArray	4, 7, 9, 11, 13, 14, 15, 16, 17, 24, 25, 26, 27 (+uArray and RNAseq)	[118,119,120,121,122]
OS	Up	1/56OS, 2/19OS (IIB, III), 3/30OS, 4/57OS, 5/53OS	SW1353, MG-63, SAOS2, HOS, U2OS	Ex2_Ex3, Ex1_Ex1/5, Ex12_Ex13, Ex5_Ex6, Ex1_Ex2	3, 4, 7, 9, 10, 11, 13, 14, 15, 16, 17, 18, 19, 20, 21, 22, 23, 24, 25, 26, 27, 28	[123,124,125,126,127,128,129]
MM	Up	1/80MM, 2/70MM	U266, MM.1S, NCI-H929	Ex3/4_Ex4, Ex12_Ex15	9, 13	[130,131]
PC	Up	10PC	LNCap, PC3, DU145	Ex12_Ex15, Ex17/18_Ex18, Ex6_Ex6/7	1, 3, 9, 10, 11, 13, 15, 16, 19, 21, 22, 24, 25, 28	[132]
ATL/AML	Up	1/178AML, 2/100AML, 3/109AML, 4/6ATL, 5/27T-ALL	MOLM-13, HL-60, MT-2, MT-4, C8166, MT-1, HPB-ATL-2, HPB-ATL-T, ED, TL-Om1, MOLT4s, CCRF-CEM, KOPT-K1	Ex1_Ex2, Ex1_Ex1, Ex17/18_Ex18	1, 4, 6, 7, 9, 10, 11, 13, 14, 15, 16, 17, 18, 19, 20, 21, 22, 23, 24, 25, 26, 27, 28	[133,134,135,136,137]
Melanoma	Up		NZM, OM431, A375	Ex1_Ex1, Ex5_Ex6	1, 3, 4, 6, 7, 9, 10, 11, 13, 14, 15, 16, 17, 18, 19, 20, 21, 22, 23, 24, 25, 26, 27, 28	[138]
	Up		NZM	Outward facing primers targeted against exons 2, 4, 6, 8, 14 and 16	CirRNA: More than 30 isoforms differentially expressed in melanoma cells	[78]
	Fusion MTAP	174 cell lines included in this study were derived from metastasized tumors of 134 melanoma patients				[139,140]
EC	Up	1/87EC, 2/20EC, 3/Transcriptome data from 552UC, 575UC	HEC-1A, RL95-2	Ex1_Ex2, Ex1_Ex1, Ex6_Ex6	3, 4, 6, 7, 9, 10, 11, 13, 14, 15, 16, 17, 18, 19, 20, 21, 22, 23, 24, 25, 26, 27, 28	[141,142,143,144]
iCCA	Up	39iCCA		Ex19_Ex20	1, 5, 8, 9, 10, 11, 21, 22, 23, 25	[145]
RC	Up	1/42KIRC, 2/108ccRCC	769-P, ACHN, 786-O, Caki-1, Caki-2, ACHN	Ex1_Ex1, Ex5_Ex6, Hs03300540_m1, HCR and RNaseq	3, 4, 6, 7, 9, 10, 11, 13, 14, 15, 16, 17, 18, 19, 20, 21, 22, 23, 24, 25, 26, 27, 28	[69,146]
RB	Up	28RB	HXO-RB44, Y79	Ex1_Ex2, Ex11_Ex15	4, 7, 9, 11, 13, 17, 25, 26, 27	[56,147]
HNSCC/LSCC	Up	1/60LSCC, 2/54LSCC, 3/28LSCC, 4/35NPC, 5/522HNSCC	Tu177, HN4, AMC-HN-8, NP69, FaDu, CAL27, CNE1, CNE2, S18, HONE1, 5–8F, AMC-HN-8, SNU-899, HNEC	Ex2_Ex3, Ex21_Ex21, Ex1_Ex1, Ex1_Ex2, Ex1/5_Ex6, uArray, RNAseq	1, 2, 3, 4, 5, 6, 7, 8, 9, 10, 11, 12, 13, 14, 15, 16, 17, 18, 19, 20, 21, 22, 23, 24, 25, 26, 27, 28	[148,149,150,151,152,153,154]
HCC	Up	1/30HCC, 2/34LC, 3/100HCC, 4/85HCC, 5/50Cirrhosis, 6/130HCC, 7/77HCC, 8/317HCC	Huh7, Hep3B, Sk-Hep1, MHCC97H, SMMC-7721, HepG2	Ex21_Ex21, Ex1_Ex1, Ex14_Ex15, Ex15_Ex16, Ex1_Ex2, Ex12_Ex15, RNAseq	1, 2, 4, 5, 6, 7, 8, 9, 10, 11, 12, 13, 14, 15, 16, 17, 18, 19, 20, 21, 22, 23, 24, 25, 26, 27, 28	[64,155,156,157,158,159,160,161,162]

(LC: lung cancer, GC: gastric cancer, BC: breast cancer, OC: ovarian cancer, CC: cervical cancer, CRC: colorectal cancer, BladC: bladder cancer, TC: thyroid cancer, BrC: brain cancer, OS: osteosarcoma, MM: myeloma, PC: prostate cancer, ATL/AML: leukemia, EC: endometrial cancer, RC: renal cancer, RB: retinoblastoma, HNSCC/LSCC: head/neck cancer, iCCA: intrahepatic cholangiocarcinoma and HCC: hepatocellular cancer).

### 6.2. MTAP-ANRIL Fusion in Cancer

The contribution of the *MTAP-ANRIL* fusion is also critical when assessing the involvement of ANRIL in cancer biology. This fusion gene, documented to exhibit a frequency exceeding 7% in melanoma, results from a chromosomal rearrangement between *ANRIL* and *MTAP*, a tumor suppressor gene involved in purine metabolism (methylthioadenosine phosphorylase) [139]. In this context, recent research has demonstrated that the *MTAP-ANRIL* gene fusion leads to the suppression of the wild-type *MTAP* expression and facilitates an epithelial–mesenchymal transition-like process through the activation of JNK and p38 MAPK pathways, both in vitro and in vivo [140].

### 6.3. Polymorphisms of ANRIL in Cancer

Since its first association with pathologies in 2007 [31], the *9p21* locus have been the topic of several genome-wide association studies (GWAS), unveiling ANRIL sequences as a hotspot for single nucleotides polymorphisms (SNPs) that are susceptibility factors for several pathologies such as CVD, T2D and cancers [163,164]. Focusing on the cancer-related pathologies, 34 SNPs were identified to be associated with higher risk of developing different types of cancer including LC, GC, BC, CC, TC, BrC, MM, PC, ATL/AML/B-ALL, melanoma/BCC, EC, ADC, HNSCC/LSCC/ESCC, PancC, OS and overall cancer risk (Table 2). In addition to this trait, SNPs are also correlated, for instance, with reduced overall survival (rs1333049) in ESCC [165], with larger tumor size (rs11333048) in TC [166], with higher TMN stage (rs3217992) in OS [167], and linked to the presence of metastases (rs1063192) in TC [168] (Table 2). Note that despite this clear association, most of the studies do not allow researchers to discern whether the observed associations reflect causality or if they indicate other underlying complexities within the biological system. This observation underscores the importance of conducting comprehensive investigations, including functional studies and mechanistic analyses similar to the exhaustive investigations conducted for instances such as rs10811656/rs10757278 in the context of CVD [52].

A total of 76% of the SNPs are localized in introns, 17% in downstream, and 6% in upstream sequences. Note that among all these SNPs, the expression of ANRIL has not been systematically evaluated, which makes the correlation between risky genotype and ANRIL expression difficult. However, it is possible to consider two levels that are likely to be affected by these variations. Firstly, at the DNA level, these variations may have an impact on the expression of the *9p21* locus through the disruption of binding sites for transcriptional regulators and/or enhancer sequences (rs10757278, rs10811656, rs4977757, rs1333045, rs1537373) as it is described for CVD [18,52,217,218]. In cancer, studies have predicted that the intronic risk allele rs17694493 disrupts both the transcription factors (STAT1 and RUNX1) and androgen receptor-binding motifs in PC. This may actively participate in the cell cycle regulation by modulating the expression of the *CDKN2B-CDKN2A* gene cluster, thereby playing a causal role in predisposing cancer risk [65,219]. As mentioned previously, the expression of the *9p21* locus also depends on the methylation state of the CpG island which modulates the association of CTCF to the ANRIL promoter [57]. One reasonably hypothesizes that the presence of SNPs may interfere with CTCF binding. In this case, this would be reminiscent of the SNP rs12936231 that has been found to disrupt the CTCF binding site within the *ZPBP2* gene in the context of asthma [220].

At the RNA level, sequence variations have the potential to impact protein binding sites and structural elements. This concept has been investigated in the context of a risky genotype associated with myocardial infarction, specifically with regard to rs10965215 and rs10738605. It is hypothesized that these variations reduce the free energy of ANRIL secondary structures which may interfere with the binding of proteins and consequently with the ANRIL activities [221]. SNPs may also disrupt the interaction between splicing factors (such as SR or hnRNPs) and regulatory elements (Exonic Splicing Enhancer ESE, Exonic Splicing Silencer ESS, Intronic Splicing Silencer ISS and Intronic Splicing Enhancer ISE) within ANRIL. This may lead to a similar effect observed for PSMD13, where the presence of the SNP rs7128029 affects its splicing pattern, leading to the exclusion of an exon in EC [222]. If so, the heterogeneity of ANRIL isoforms observed in pathological situations may be attributed to the presence of peculiar SNPs.

## 7. ANRIL Activities

### 7.1. Cytoplasmic Activities of ANRIL

**Figure 3:**
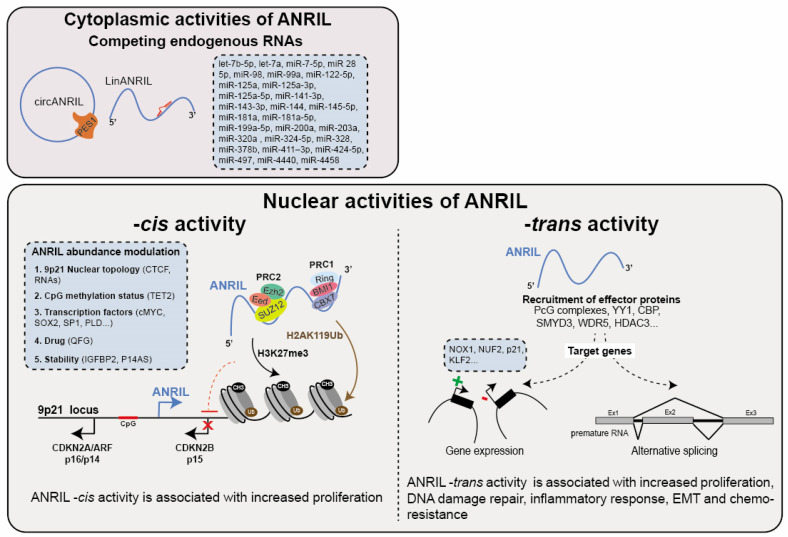
Functions of ANRIL. ANRIL exerts various regulatory roles both in the cytoplasm and nucleus. In the cytoplasm, ANRIL (circANRIL: circular ANRIL and linANRIL: linear ANRIL) acts as a competing endogenous RNA (ceRNA) for miRNAs and proteins, thereby modulating gene expression at the post-transcriptional level. Within the nucleus, ANRIL functions as a *cis*-regulator, facilitating the recruitment of Polycomb group proteins (PcG) to the *9p21* locus. This leads to transcriptional repression of genes including *CDKN2B*, achieved through the deposition of H2AK119ub (PRC1) and H3K27me3 (PRC2) histone marks at the *9p21* locus. Furthermore, ANRIL modulates gene expression at the chromatin level in *-trans* by guiding the recruitment of chromatin modifiers or transcriptional activators (such as PcG, YY1…) to specific loci (e.g., *NOX1*, *KLF2*…). Emerging evidence indicates also that ANRIL impacts alternative splicing patterns. Overall, these regulatory activities predominantly enhance cell proliferation, migration, invasion, and metastasis while suppressing apoptosis and senescence, primarily attributed to the modulation of key cancer-related gene expression.

The association between cancer biology and the cytoplasmic isoforms of ANRIL primarily stems from their capacities to function as competing endogenous RNAs (ceRNA). This functionality arises from ANRIL’s ability to form hybrid complexes with miRNAs when it is overexpressed in cancer cells. Consequently, this interaction results in the decreased activity of tumor-suppressor microRNAs, ultimately leading to the upregulation of genes that are normally repressed by these miRNAs (Table 3). To date, the proposed ceRNA role of ANRIL involves a single interaction with a total of 28 miRNAs which hybridize with 10 ANRIL exons predominantly occurring on exons 1, 11, and 20 (let-7b-5p, let-7a, miR-7-5p, miR-28-5p, miR-98, miR-99a, miR-122-5p, miR-125a, miR-125a-3p, miR-125a-5p, miR-141-3p, miR-143-3p, miR-144, miR-145-5p, miR-181a, miR-181a-5p, miR-199a-5p, miR-200a, miR-203a, miR-320a, miR-324-5p, miR-328, miR-378b, miR-411–3p, miR-424-5p, miR-497, miR-4440, miR-4458) (Figure 3). The potential ANRIL/miRNA associations have been correlated with multiple cancers including GC, BC, OC, CC, CRC, TC, BrC, OS, PC, MM, PC, ATL/AML, EC, RC, RB, HNSCC/LSCC, HCC, LC and some are even associated with higher TMN stage (Table 3). These observations strongly support the role of ANRIL as an oncogene by virtue of its ceRNA activity. For instance, this activity has been implicated in modulating the miR-320a/HMGB1 axis, a pathway associated with inflammation in TC [223]. Moreover, the interactions between ANRIL and let-7 miRNA (7a, 7b-5p), miR-122-5p and miR-181a have been shown to increase cancer stem cell proliferation and epithelial–mesenchymal transition (EMT) by affecting multiple pathways including Wnt, NOTCH, STAT3/NF, MAPK/ERK, PI3K/AKT, and glycolysis in BrC, OC, TC, CR and HNSCC [106,116,120,151,152,224]. In the context of the let-7 miRNAs, ANRIL competitively interacts with pivotal components within the aforementioned pathways, such as the TCF-4, CCND1, HMGA2, NUMB, and c-Myc mRNAs. This counteracts their role as tumor suppressors, thus hampering their inherent tumor-suppressive functionality [151,224]. Additionally, ANRIL positively influences cell proliferation through the miR-181a-5p/CCNG1, miR-203a/CDK2, miR-141-3p/CCND1/2, and miR-497/CDK6 axes in GC, HCC, RC, and HNSCC, respectively [96,146,225,226].

Interestingly, the ceRNA activity of ANRIL is associated with chemoresistance. It has been shown that ANRIL acts as ceRNA for the miR-125a in the development of TNBC and its chemoresistance of doxorubicin by enhancing glycolytic activity, specifically targeting enolase 1 (ENO1) [99]. ANRIL is also likely to act on the multidrug transporters miR-125a-5p/MRP4 axis, which are important players in EC paclitaxel resistance [141]. In the context of CRC, ceRNA activity of ANRIL has been proposed to promote resistance to 5-Fluorouracil by inhibiting the ABCC1/let-7a pathway [247]. Finally, ANRIL may favor cisplatin resistance in LC and OS by modulating the miR-328/ABCG2/MDR1 and miR-125a-5p/STAT3 signaling pathways, respectively [125,228].

Radiotherapy is an important treatment for cancer, mainly by triggering DNA double-strand breaks to induce cell death. Promoting DNA damage repair can decrease the radiosensitivity of tumor cells. Since ANRIL has been described to be a key regulator in DNA damage repair (HDR), it is well suited to play a role in this process. In agreement, ANRIL positively acts on HDR by modulating the miR-7-5p/PARP1/RAD51 and miR-145-5p/MMP1 pathways in LC and TNBC respectively [100,228].

Although the majority of studies do not distinguish between circular and linear isoforms of ANRIL, and only a limited number of them assess the functional interactions between ANRIL and miRNAs through techniques such as dual-luciferase assays, these findings emphasize the complex regulatory interplay between ANRIL, miRNAs and cancer.

### 7.2. Nuclear Activities of ANRIL

#### 7.2.1. -*cis* Activity

ANRIL functions as a *cis*-regulator by facilitating the recruitment of the Polycomb group proteins (PcG) to the *9p21* locus. This results in the transcriptional repression of, at least, the *CDKN2B* gene through the deposition of H2AK119ub (PRC1) and H3K27me3 (PRC2) histone marks at the promoter region of the *CDKN2A*/*ARF-ANRIL* genes in PC and normal lung fibroblast (Figure 3) [25,248]. While the exact molecular mechanism and sequential elements involved in this association remain incompletely understood, the recruitment of CBX7 (PRC1) by ANRIL appears to depend on a stem-loop structure [25]. Note that this activity has been attributed to the linear isoforms of ANRIL. Conversely, a particular circular isoform has been identified to interact with EZH2 (a component of PRC2), leading to the displacement of EZH2 from the *CDKN2A*/*ARF-ANRIL* promoter. This alteration in EZH2 localization influences the deposition of H3K27me3 on the latter, resulting in the increased expression of ANRIL in human primary fibroblasts undergoing oncogene-induced senescence [249].

An intriguing aspect that remains to be fully understood is the mechanism through which ANRIL interacts with protein binders to carry out its functions. Notably, it has been demonstrated that the association between ANRIL and PRC1 relies on chaperone proteins such as MOV10, whose helicase activity is essential for recruiting the ANRIL/PRC1 complex to the CDKN2A/ARF promoter [250]. One hypothesis suggests that MOV10 might influence the structural conformation of ANRIL, promoting its interaction with the PRC1. If so, it would be reminiscent of the lncRNA roXes (RNA on the X) involved in dosage compensation in Drosophila melanogaster. In this context, the RNA helicase MLE known to be crucial for unwinding conserved stem-loop structures facilitates the association between the roXes and MSL2, a critical protein involved in the assembly and regulation of the MSL complex [251].

Another point which also remains to be elucidated is how ANRIL associates the genome to exert its functions at specific loci. It has been proposed that the -*cis* activity depends on the formation of a triple helix via a triplex-forming oligonucleotide (TFO) located in ANRIL Exon1 and the CDKN2B promoter [252]. Triplexes are formed when a single-stranded RNA fragment accommodates the major groove of the double stranded DNA. Since this association relies on base-pairing interactions, such as Hoogsteen or reverse Hoogsteen hydrogen bonds occurring between ANRIL and the CDKN2B promoter, such an anchoring mechanism may explain, at least in part, how ANRIL recognizes specifically the *9p21* locus [253].

#### 7.2.2. *-trans* Activity of ANRIL

##### ANRIL Regulation of Genes Located Outside the 9p21 Locus

Several studies have provided evidence suggesting that ANRIL exerts direct regulation on gene expression beyond the *9p21* locus (Figure 3). Indeed, RNA-FISH experiments performed in PC and EC have demonstrated that ANRIL localization is not restricted to the *9p21* locus [25,254]. The overexpression of ANRIL fragments in HEK293 cells has shown to upregulate 219 genes and downregulate 708 genes involved in processes such as development, adhesion, proliferation, and apoptosis [19]. In a more recent transcriptomic analysis of vascular smooth muscle cells (VSMCs), Lo Sardo et al. identified altered expression profiles in approximately 3000 genes located outside the *9p21* locus upon depletion of the 3′ end of the *ANRIL* gene compared to wild-type cells again involved in proliferation and apoptosis [255]. To distinguish between direct and indirect genomic targets of ANRIL, Alfeghaly and colleagues combined ChIRP-seq genomic occupancy data with transcriptomic analysis in ANRIL-knockdown HEK293 cells. This analysis could identify 123 downregulated and 65 upregulated genes as direct targets of ANRIL [20]. Similar to its -*cis* activity, ANRIL is thought to exert its repressive -*trans* activity by interacting with Polycomb group proteins (PcG). Consistent with this notion, ANRIL is capable of epigenetically suppressing the transcription of the *p21* and *KLF2* (Kruppel-like factor 2) genes in HCC and LC. This repression occurs through ANRIL binding to the PRC2 complex and subsequently to its recruitment to the promoters of these genes [64,256]. It is important to note that among the direct target genes found to be silenced in the study conducted by Alfeghaly (2021), only 17% of them are likely to be regulated through a PcG-dependent mechanism [20]. This finding suggests that ANRIL may employ PcG-independent mechanisms to silence gene expression. Further in-depth investigation is needed to fully understand these mechanisms.

As previously mentioned, ANRIL has the potential to activate gene expression. According to Zhou et al., they elucidated the mechanism through which the ANRIL/YY1 complex contributes to the activation of *IL6* and *8* (interleukins 6 and 8) genes in HUVEC cells in response to a TNF-α pro-inflammatory signal [26]. Furthermore, ANRIL likely enhances the growth, migration, and invasion abilities of HNSCC and TC cancer cells by positively regulating the TGF-β1/Smad signaling pathway [257,258]. Regarding its role in RC, ANRIL recruits the CBP (CREB-binding protein) and SMYD3 (SET and MYND domain-containing 3) epigenetic-modifying complex to activate the transcription of *NUF2* at the chromatin level. This activation occurs through the deposition of local modifications of H3K27ac and H3K4me3 histone marks [69]. Moreover, ANRIL was found to associate with WDR5 and HDAC3 proteins in human aortic smooth muscle cells (HASMC) [259]. This complex is necessary for the deposition of H3K9Ac and H3K4me3 histone marks on the promoter region of the *NOX1* gene, resulting in its transcriptional activation.

Note that approximately 24% of the miRNAs identified as ANRIL targets in the context of its ceRNA activity do not exhibit apparent hybridization sites on ANRIL (miR-99a, miR-125a-3p, miR-144, miR-145-5p, miR-200a, miR-203a, miR-384) [94,100,121,225,231,237,239,243,245]. This observation suggests the possibility of indirect effects of ANRIL on the miRNA abundance or may highlight the involvement of ANRIL *-trans* activity in epigenetically down-regulating the expression of these miRNAs. Such mechanisms have been already reported in various contexts, including HCC, where ANRIL enhances cancer migration and proliferation by modulating the expression of the miR-191 and by subsequently affecting the vimentin expression [260]. Additionally, ANRIL has been found to epigenetically silence miR-99a and miR-449a via PcG complexes, promoting the CDK6/E2F1 pathway and establishing a positive feedback loop for its own expression, continuing, therefore, to promote gastric cancer cell proliferation [98]. The mechanism engaged by ANRIL for specifically associating the genomic loci in the context of its *-trans* activity is not fully understood yet. However, the ChIRP-seq data generated by Alfeghaly and colleagues allowed for the identification of 3227 binding sites across the genome in HEK293 cells. Interestingly, among the sequences of these binding sites, 98% are enriched with G/A repeats. Moreover, ANRIL exon 8, which is 70% covered by the subcategory of LTR named ERVL-MaLR, is involved in its genomic occupancy in HEK293 cells by forming triple helices for instance [20]. However, this genome recognition mode cannot solely explain ANRIL binding to the chromatin, and alternative modes engaged by ANRIL to associate with the genome have to be considered. LncRNA–chromatin recognition can take place through the interaction with specific protein partners that serve as bridge between the DNA and the lncRNA as the heterogeneous nuclear RiboNucleoProtein U (hnRNP U) matrix protein [261,262]. Another mechanism relies on the direct interaction of the lncRNA with the DNA molecule via RNA–DNA hybrid duplexes formed via canonical Watson–Crick base-pairing. The resulting hybrid is named R-loop [263,264]. Further investigations are needed to identify potential R-loops or ANRIL protein-binders involved in its genomic association.

##### Role of ANRIL in Modulating the Alternative Splicing

Genomic occupancy assays performed in HEK293 cells revealed that the majority of the binding sites for ANRIL are located in non-coding regions, including introns and intergenic regions. Remarkably, 40.3% of the ANRIL sites are intronic, and, more importantly, 24% of the genes contacted by ANRIL in HEK293 are affected in terms of alternative splicing (AS) upon ANRIL knockdown [20]. In addition, the overexpression of ANRIL in HUVEC cells, coupled with RNA-seq and AS analysis, revealed significant impacts on the AS patterns of numerous genes. These affected genes play essential roles in translation, DNA repair, RNA processing, and participate in the NFκB signaling pathway [27]. These findings highlight the significant influence of ANRIL on the AS landscape (Figure 3). It is noteworthy that this activity is reminiscent of the lncRNA asFGFR2 described to act on the AS of the FGFR2 transcript [265]. In this case, asFGFR2 modulates the chromatin structure at the FGFR2 locus, resulting in the limitation of the binding of the MRG15-PTB chromatin adapter complex to exon IIIb. As a consequence, this modulation promotes the inclusion of exon IIIb in the FGFR2 mRNA. Interestingly, this isoform plays an antitumoral role in HCC, which definitively links lncRNA, AS modulation and cancer fields in a concrete manner [266].

Cancer-specific splicing events have been detected in diverse cancer types such as BC, LC, PC, and AML/ALL [15,267,268]. The combinatorial nature of the AS relies on the selective utilization of splicing sites within the pre-mRNA, which is mediated by factors including RNA Binding Proteins (RBPs) such as the SR and hnRNP proteins, which function as *-trans* acting regulatory factors [269]. In addition, a close association between chromatin characteristics and AS has been identified. Nucleosomes, through their specific patterns of histone modifications, have the ability to influence AS [270]. For instance, exons exhibit a higher occurrence of certain histone modifications such as H3K27me2, H3K36me3, and H4K20me3. The presence of these modifications can impact AS by interacting with protein factors that facilitate the recruitment of *trans*-acting regulatory factors, or even directly recruiting components of the spliceosome [271,272,273,274,275,276,277]. Even though the specific molecular mechanism employed by ANRIL to regulate AS remains unknown, its ability to associate with proteins and modulate chromatin landscape highly suggests that ANRIL plays a role in the AS process in a direct manner. If so, one reasonably hypothesizes that this capacity might also contribute to ANRIL involvement as a susceptibility factor in cancer development.

## 8. Conclusions

In conclusion, ANRIL is a critical factor in maintaining the balance of cellular functions despite its relatively simple nature. It plays a crucial role in regulating the progression of the cell cycle by interacting with PcG and guiding them to specific genomic loci, resulting in the silencing of genes like *CDKN2B*. Additionally, ANRIL acts as a ceRNA, impacting the activity of over 20 miRNAs involved in various cellular processes such as cell proliferation, invasion, inflammation, and EMT. Consequently, the dysregulation of ANRIL disrupts normal cell cycle control, leading to uncontrolled cell growth and the development of cancer. This explains why ANRIL is found to be upregulated in more than 20 types of cancer, as well as in conditions like CVD and T2D. To the best of our knowledge, ANRIL is the lncRNA most strongly associated with these pathological conditions.

While substantial progress has been made in unraveling the functional roles of ANRIL in cancer, there is still much to discover. The precise molecular mechanisms underlying ANRIL activities, particularly its PcG-independent functions and its role on the splicing modulation, remain incompletely understood. In addition, the complexity of ANRIL isoforms allows for versatility in its interactions with different proteins, RNA molecules, and genomic regions, thereby influencing various biological processes. The investigation of the contribution at the molecular level of each isoform individually represents a major challenge in the field. Thus, ANRIL stands as a fascinating and multifaceted molecule with diverse implications in cancer biology. Its dysregulation makes it a promising area for continued investigation and the development of innovative therapeutic approaches.

Since ANRIL is predominantly overexpressed, a potential therapeutic approach could involve reducing its intracellular abundance within cancerous lesions. This could be achieved through methods such as RNA interference (RNAi), antisense oligonucleotides (ASOs), the RNA-targeting CRISPR-Cas system, or epigenetic modulators employed to alter the epigenetic profile of the ANRIL gene [278]. Another strategy could involve utilizing small molecules designed to hinder ANRIL functions, achieved through modifications of lncRNA–protein interactions or the induction of structural changes [279]. It is worth noting that the primary challenge lies in crafting a molecule that guarantees effectiveness while minimizing unintended effects. The creation of such a molecule relies on a comprehensive understanding of the lncRNA activities at the molecular level, which still need to be thoroughly elucidated for ANRIL. Also, given the complexity of ANRIL interactions and effects, combination therapies that target multiple aspects of its activity could be more effective than single-target approaches. In the end, this could lead to the development of personalized treatments, enabling the adaptation of strategies for reducing or inhibiting the expression patterns or activities, respectively, of specific ANRIL isoforms in cancers.

The key role of lncRNAs in cancer development and progression has made these classes of RNA a major point of interest for researchers. As ANRIL, many are found overexpressed in cancer tissues or cancer lines in culture. Hence, the advancements in ANRIL biology should provide knowledge about its multiple activities but may also lead to the discovery of more generic activities attributed to lncRNAs.

## Figures and Tables

**Table 2:** Summary of the SNPs found within the ANRIL gene and their connection to various types of cancers. The summary provides details about the specific cancer types involved and includes the relevant references.

Cancers SNPs	LC	GC	BC	TC	BrC	MM	PC	ATL/AML/ B-ALL	Melanoma/ BCC	EC	HNSCC/LSCC/ ESCC	PancC	OS	Overall Cancer
rs2151280	[169]				[170,171]	[172]		[173]	[174]					
rs17694493		[175]												
rs4977574				[166]			[176]							[177]
rs78545330			[178,179]											
rs1333040	[180]													
rs10757278							[176,181]							[177]
rs62560775	[182]		[182]											
rs1011970			[183,184,185,186,187]				[182]		[163]					
rs2157719					[188,189,190]						[191]			
rs4977756	[192]				[190,193,194,195,196,197,198,199,200]						[201]			
rs634537					[188,202]									
rs2811712			[163]					[203]						
rs1333048				[166]			[176,181]							[177]
rs564398								[204]	[205]	[206]	[206]			
rs10965215			[178]						[205]					
rs1412832												[207]		
rs2518723			[178]											
rs77792598			[178]											
rs4977753			[178]											
rs75917766			[178]											
rs1333049	[208]		[209]								[165]			
rs8181047											[210]			
rs1412829					[190,193,197,200,211]						[201,212,213]			
rs145929329					[188]									
rs3218005			[163,214]											
rs3217992								[203]				[215]	[167]	
rs2811709								[203]						
rs518394									[205]					
rs7857345											[216]			
rs1063192				[168]	[163,190]						[191,201]			
rs615552										[206]	[206]			
rs573687										[206]	[206]			
rs10811661											[165]			
rs10120688	[208]													

(LC: lung cancer, GC: gastric cancer, BC: breast cancer, TC: thyroid cancer, BrC: brain cancer, MM: myeloma, PC: prostate cancer, ATL/AML/B-ALL: leukemia, Melanoma/BCC: melanoma/basal cell carcinoma, EC: endometrial cancer, ADC: OC: ovarian cancer, CC: cervical cancer, CRC: colorectal cancer, BladC: bladder cancer, BrC: brain cancer, OS: osteosarcoma, MM: myeloma, PC: prostate cancer, ATL/AML: leukemia, EC: endometrial cancer, HNSCC/LSCC/ESCC: head/neck cancer, PancC: pancreas cancer, OS: osteosarcoma).

**Table 3:** Summary detailing the connection between miRNAs and ANRIL. It covers various aspects such as the types of cancer involved, the influence of ANRIL/miRNA interactions on different cancer-related processes, the target genes, the specific location of hybridization on ANRIL, and the relevant references.

miRNAs	Cancers	Effect of ANRIL/miRNA Axis	miRNA Target	Hybridization Location on ANRIL	References
let-7b-5p	BrC	Promotes proliferation, migration and suppresses apoptosis. Enhances tumor growth in mice xenograft.	-	exon 11	[120]
let-7a	OC, CRC, PC HNSCC	Promotes proliferation, migration and suppresses apoptosis. Enhances 5-FU, Oxaliplatin, and Cisplatin chemoresistance and tumor growth in mice xenograft. High expression associated with higher clinical stage patient.	HMGA2, ABCC1	exon 11	[106,151,227]
miR-7-5p	LC, ATL/AML	Promotes cell viability, migration and invasion. Suppresses radiosensitivity through enhancing HDR system. Enhances tumor growth in mice xenograft.	TCF4 PARP1 (down-regulated genes BRCA1/RAD51)	exon 1	[137,228]
miR-28-5p	CRC	Promotes proliferation.	URGCP	exon 2	[229]
miR-98	LC	Enhances proliferation and cisplatin chemoresistance.	-	exon 11	[230]
miR-99a	HNSCC, GC	Promotes proliferation, migration, invasion and suppresses apoptosis.	BMI1	-	[94,231]
miR-122-5p	TC, HCC, BC	Promotes proliferation, migration, invasion and tumor growth in vivo. Suppresses apoptosis and enhances tumor in mice xenograft.	P4HA1 STK39	exon 11	[116,232,233]
miR-125a	BC, HNSCC, MM	Promotes proliferation, migration, and invasion. Suppresses apoptosis and radiosensitivity. Enhances doxorubicin chemoresistance and tumor growth in mice xenograft. High expression associated with advanced ISS stage, and decreased complete response.	ENO1 ESRRA β2-MG	exon 1	[99,234,235,236]
miR-125a-3p	OC	Promotes proliferation, migration and suppresses apoptosis.	p38	-	[237]
miR-125a-5p	LC, OS, EC	Promotes proliferation and suppresses apoptosis. Enhances ubenimex, cisplatin and paclitaxel chemoresistance.	APN STAT3 Bcl2, MRP4	exon 4	[125,141,238]
miR-141-3p	RC	Promotes proliferation, migration and invasion and suppresses apoptosis. Enhances tumor growth in mice xenograft.	CCND1 and CCND2	exon 3	[146]
miR-143-3p	OC	Promotes proliferation, migration and invasion, and suppresses apoptosis. Enhances tumor growth in mice xenograft.	SMAD3	exon 1	[105]
miR-144	HCC	Promotes proliferation, migration invasion, and suppresses apoptosis.	PBX3	-	[239]
miR-145-5p	BC	Involved in the development of early breast cancer.	MMP1	-	[100]
miR-181a	HCC, PancC	Promotes proliferation, migration and invasion.	HMGB1, Snai2	exon 1	[152,240]
miR-181a-5p	CRC, CC	Promotes proliferation, migration and invasion. Suppresses apoptosis, cell cycle arrest and senescence. High expression suppresses chitooligosaccharide (COS)-related radiosensitivity.	CCNG1, TGF-β1	exon 1	[96,241,242]
miR-199a-5p	HCC, BrC	Promotes proliferation, migration and invasion. Suppresses apoptosis and cell autophagy. Enhances mitochondrial function and tumor growth in mice xenograft.	DDR1 ARL2	exon 17, 20	[118,156,158]
miR-200a	BrC	Promotes proliferation and suppresses apoptosis.	-	-	[243]
miR-203a	HCC, BrC	Promotes proliferation and inhibits anoikis.	Bcl-2, p-AKT	-	[121,225]
miR-320a	TC	Promotes proliferation, migration and invasion, and suppresses apoptosis. Enhances tumor growth in mice xenograft.	HMGB1	exon 18	[223]
miR-324-5p	HNSCC	Promotes proliferation and suppresses apoptosis. High expression associated with advanced clinical stage, metastasis.	ROCK1	exon 20	[148]
miR-328	RB	Enhances proliferation and cisplatin chemoresistance.	ABCG2, MDR1	exon 20	[56]
miR-378b	LC	Promotes proliferation, migration and invasion, and suppresses apoptosis.	NR2C2	exon 19	[244]
miR-384	HCC	Promotes proliferation, migration, invasion and suppresses apoptosis and cell cycle arrest.	STAT3	-	[245]
miR-411-3p	MM, OC	Promotes proliferation, migration invasion, and suppresses apoptosis. Enhances tumor stem cell-like property and tumor growth in vivo mice xenograft.	HIF-1α	exon 21	[108,131]
miR-424-5p	HCC	Enhances cell viability, migration and invasion.	-	exon 17	[155]
miR-497	HNSCC	Promotes proliferation, migration, invasion, and suppresses apoptosis.	CDK6	exon 17	[226]
miR-4440	BC	Promotes proliferation, migration and invasion.	-	exon 2	[178]
miR-4458	OS	Promotes proliferation, migration, and invasion. Enhances tumor growth in mice xenografts.	MAP3K3	exon 11	[246]

(LC: lung cancer, GC: gastric cancer, BC: breast cancer, TC: thyroid cancer, BrC: brain cancer, MM: myeloma, PC: prostate cancer, ATL/AML/B-ALL: leukemia, Melanoma/BCC: melanoma/basal cell carcinoma, EC: endometrial cancer, ADC: OC: ovarian cancer, CC: cervical cancer, CRC: colorectal cancer, BladC: bladder cancer, BrC: brain cancer, OS: osteosarcoma, MM: myeloma, PC: prostate cancer, ATL/AML: leukemia, EC: endometrial cancer, HNSCC/LSCC/ESCC: head/neck cancer, PancC: pancreas cancer, OS: osteosarcoma).

## Data Availability

The data presented in this study are available in this article.

## Abbreviations

ABCC1: ATP-binding cassette sub-family C member 1, ANRIL: Antisense Non-coding RNA in the INK4 Locus, ACHN: Adenocarcinoma Human Kidney, ARF: Alternative Reading Frame, AUF1: AU-rich Element RNA-binding Protein 1, CBX7: Chromobox Homolog 7, CC: Cervical Cancer, CCA: Cholangiocarcinoma, CCND1/2: Cyclin D1/D2, CDK6: Cyclin-dependent Kinase 6, CDKN2A-2B: Cyclin-dependent Kinase Inhibitor 2A-2B, c-MYC: Cellular Myelocytomatosis Oncogene, CTCF: CCCTC-binding Factor, CVD: Cardiovascular Disease, CXCL11: C-X-C Motif Chemokine Ligand 11, E2F1: E2F Transcription Factor 1, EMT: Epithelial–mesenchymal Transition, ERK: Extracellular Signal-Regulated Kinase, ERVL-MaLR: Endogenous Retrovirus-Like MaLR, ERα: Estrogen Receptor Alpha, ESE: Exonic Splicing Enhancer, ESS: Exonic Splicing Silencer, EZH2: Enhancer of Zeste Homolog 2, EZR: Ezrin, FGFR2: Fibroblast Growth Factor Receptor 2, FISH: Fluorescence In Situ Hybridization, GAS5: Growth Arrest-Specific 5, GC: Gastric Cancer, GTEx: Genotype-Tissue Expression, GWAS: Genome-Wide Association Study, H3K27ac: Histone H3 Lysine 27 Acetylation, H3K27me2: Histone H3 Lysine 27 Dimethylation, H3K36me3: Histone H3 Lysine 36 Trimethylation, H3K37me3: Histone H3 Lysine 37 Trimethylation, H3K4me3: Histone H3 Lysine 4 Trimethylation, H3K9ac: Histone H3 Lysine 9 Acetylation, H4K20me3: Histone H4 Lysine 20 Trimethylation, HCC: Hepatocellular Carcinoma, HIF-1α: Hypoxia-Inducible Factor 1 Alpha, HMGB1: High Mobility Group Box 1, hnRNP U: Heterogeneous Nuclear Ribonucleoprotein U, HNSCC/LSCC: Head and Neck Squamous Cell Carcinoma/Lung Squamous Cell Carcinoma, HOTAIR: HOX Transcript Antisense Intergenic RNA, IFNA21: Interferon Alpha 21, IFN-γ: Interferon Gamma, IGF2BP3: Insulin-like Growth Factor 2 mRNA-Binding Protein 3, INK4: Inhibitor of Cyclin-Dependent Kinase 4, IPF: Idiopathic Pulmonary Fibrosis, ISE: Intronic Splicing Enhancer, ISS: Intronic Splicing Silencer, JNK: c-Jun N-terminal Kinase, KLF2: Kruppel-Like Factor 2, LC: Lung Cancer, LINE: Long Interspersed Nuclear Element, lncRNAs: Long Non-Coding RNAs, LTR: Long Terminal Repeat, LUAD: Lung Adenocarcinoma, MAPK: Mitogen-Activated Protein Kinase, MDR1: Multidrug Resistance Protein 1, miRNAs: MicroRNAs, MLE: Maleless, MM: Myeloma, MMP1: Matrix Metallopeptidase 1, MOV10: Moloney Leukemia Virus 10, MRG15: Mortality Factor on Chromosome 4 Gene 15, MRP4: Multidrug Resistance-Associated Protein 4, MSL2: Male-Specific Lethal 2, MTAP: Methylthioadenosine Phosphorylase, NF-kB: Nuclear Factor-kappa B, NOTCH: Neurogenic Locus Notch Homolog Protein, NOX1: NADPH Oxidase 1, NSCLC: Non-Small Cell Lung Cancer, NUF2: NDC80 Kinetochore Complex Component Nuf2, OC: Ovarian Cancer, OS: Osteosarcoma, PARP1: Poly(ADP-ribose) Polymerase 1, PC: Prostate Cancer, PancC: Pancreatic Cancer, PcG: Polycomb Group, PES1: Pescadillo Ribosomal Biogenesis Factor 1, PLD: Phospholipase D, PRC1: Polycomb Repressive Complex 1, PRC2: Polycomb Repressive Complex 2, PSMD13: 26S Proteasome Non-ATPase Regulatory Subunit 13, PTB: Polypyrimidine Tract-Binding Protein, QFG: Qingjie Fuzheng Granule, Rb: Retinoblastoma Protein, RB: Retinoblastoma, RBP: RNA-Binding Protein, RC: Renal Cancer, RIDL: Repeat Insertion Domains of LncRNAs, RNP: Ribonucleoprotein, roXes: RNA on X, RTqPCR: Reverse Transcription Quantitative Polymerase Chain Reaction, RUNX1: Runt-Related Transcription Factor 1, SINE: Short Interspersed Nuclear Element, SMAD3/4: SMAD Family Member 3/4, SMYD3: SET and MYND Domain-Containing Protein 3, SNPs: Single-Nucleotide Polymorphisms, SOX2: SRY-Box Transcription Factor 2, SP1: Specificity Protein 1, SR: Splicing Regulator, SRSF3: Serine/Arginine-Rich Splicing Factor 3, STAT1: Signal Transducer and Activator of Transcription 1, T2D: Type 2 Diabetes, TAD: Topologically Associated Domain, TC: Thyroid Cancer, TE: Transposable Element, TET2: Tet Methylcytosine Dioxygenase 2, TFO: Triplex Forming Oligonucleotide, TGF-β1: Transforming Growth Factor Beta 1, TMEM106B: Transmembrane Protein 106B, TNBC: Triple-Negative Breast Cancer, TNF-α: Tumor Necrosis Factor Alpha, VSMC: Vascular Smooth Muscle Cell, Wnt: Wingless/Integrated, YY1: Yin Yang 1, ZPBP2: Zona Pellucida-Binding Protein.
